# Role of Anxiety and Depression on the Interplay Between Disorders of Gut-Brain Interaction and Eating Disorders

**DOI:** 10.14309/ctg.0000000000001025

**Published:** 2026-06-11

**Authors:** Stella-Maris C. Egboh, Natasha A. Koloski, Michael P. Jones, Ayesha Shah, Gerald Holtmann, Nicholas J. Talley

**Affiliations:** 1 College of Health, Medicine and Well-Being, University of Newcastle, Callaghan, New South Wales, Australia;; 2 Department of Internal Medicine, Gastroenterology Division, Federal Medical Centre, Yenagoa, Nigeria;; 3 Department of Gastroenterology & Hepatology, Princess Alexandra Hospital, Brisbane, Queensland, Australia;; 4 Faculty of Medicine, University of Queensland, St. Lucia, Queensland, Australia;; 5 School of Psychological Sciences, Macquarie University, Sydney, New South Wales, Australia.

**Keywords:** eating disorders, disorders of gut-brain interaction, anxiety, depression, psychological distress

## Abstract

**INTRODUCTION:**

Disorders of gut-brain interaction (DGBI) are frequently encountered among patients with eating disorders (ED). Underlying both ED and DGBI may be psychological comorbidities, but it is uncertain how often psychological disorder explains the development of either disorder. We hypothesized a DGBI may predispose to a new onset ED. We aimed to determine the associations and chronological presentation of DGBI, ED, and psychological comorbidities.

**METHODS:**

Data were collected from a retrospective cohort of n = 1,256,331 patients attending general practices in the UK; 1,018 patients diagnosed with both a DGBI and an ED were included, with 777 also having data on anxiety and/or depression. Timing of order of onset and potential moderators of the sequence of occurrence were considered. Clinical significance level was set at *P* < 0.01 and odds ratio (OR) ≥ 2.0.

**RESULTS:**

Prevalence of any ED was 0.7%. Chronic constipation was associated with anorexia nervosa (OR = 2.12; 95% confidence interval 1.84–2.45) and any ED (OR = 2.06; 95% confidence interval 1.79–2.36). Irritable bowel syndrome and functional dyspepsia were significantly associated with one or more ED. More patients in primary care were diagnosed with an ED (59.5%, n = 606) preceding a DGBI than vice versa (40.5%, n = 412, *P* < 0.001). Independent predictors of having a diagnosed DGBI preceding an ED were younger age at first diagnosis of DGBI (*P* < 0.001), antecedent depression (*P* < 0.003), and antecedent proton pump inhibitor use (*P* < 0.001).

**DISCUSSION:**

Psychological disorders, ED, and DGBI overlap. ED more frequently preceded the onset of a DGBI diagnosis; younger age, antecedent depression, and proton pump inhibitor use were independently associated with a DGBI predicting an ED.

## INTRODUCTION

Disorders of gut-brain interaction (DGBI) are common gastrointestinal (GI) conditions driven by complex psychobiological processes arising from interactions of multiple systems at the gut and brain levels (1). Eating disorders are a group of uncommon mental health illnesses characterized by specific disturbances of eating behaviors (2–4). Both DGBI and eating disorder (ED) may occur together but form a complex interrelationship (3); in clinical practice DGBI may occur first and later an ED develops, whereas in other cases the ED seems to arise first often in teenage years and later DGBI emerges (5).

This relationship between ED and DGBI may be explained by the strong communication between the gut microbiota metabolites and the hypothalamus-pituitary-adrenal (HPA) axis, through the microbiota-gut-brain axis (6,7). Dietary restriction and starvation associated with ED leads to luminal microbial dysbiosis which may further drive the onset or progression of DGBI (8). Likewise, abdominal distension, a common symptom of DGBI, may increase body dysmorphia leading to disordered eating practices (3). Furthermore, early satiety (inability to finish a normal-sized meal because of the discomfort induced) is a common DGBI symptom often linked to failure of the gastric fundus to relax, and theoretically could lead to an avoidant restrictive food intake disorder (ARFID) or a classic ED (9). This complex relationship between ED and DGBI can likely be influenced by other factors including female sex, younger age, lower quality of life, and severity of GI symptoms (10). Despite the high coexistence of ED with DGBI, diagnosis is often challenging because patients may deny ED symptoms despite losing weight (3).

Anxiety and depression can potentiate the initiation and progress of both ED and DGBI through the disruption of normal physiological and behavioral function driven by the activation of the hypothalamic-pituitary axis (11,12). Anxiety and mood disorders including depression and bipolar disorder can either precede or coexist with ED and with DGBI (13,14). Hence, it is uncertain if DGBI directly predispose to one or more ED or are a consequence of the ED, and it is unclear if anxiety and depression causes both ED and DGBI, or in these conditions anxiety and depression is simultaneously present but not causal (i.e. only a comorbidity).

Understanding the impact of psychological factors on the relationship between ED and DGBI could suggest the use of psychological interventions that target the brain-gut axis in patients with coexisting disorders. Therefore, we aimed in a large sample of primary care patients to test if DGBI are associated with ED, irrespective of anxiety and depression, determine the proportion of individuals with the diagnosis of a DGBI who were later diagnosed having an ED and vice versa, and identify the specific factors moderating the chronological pattern of occurrence of these disorders. We hypothesized having a DGBI may predispose to developing a new onset ED such as bulimia nervosa or binge eating, and depression and/or anxiety would be an important risk factor.

## METHODS

### Data acquisition

The Health Improvement Network (THIN) provides a large database of anonymized electronic medical records collected at general practices and has been found to be broadly representative of the demographics of the UK (15). All recorded clinical data within THIN are coded with read codes for medical diagnosis and drug codes for prescribed medications. GI disorder diagnoses from THIN have been reported to be valid (16). Ethical approval was given by the UK National Health Services South-East Multi-Centre Research Ethics Committee (Ref: 20/SC/0011; March 20, 2020). Data were collected from a retrospective cohort of 1,256,331 patients attending general practices in the UK with a DGBI (irritable bowel syndrome [IBS], functional dyspepsia [FD], or chronic constipation) after excluding inflammatory bowel disease, celiac disease, peptic ulcer, gastric and colorectal cancer. The patient sample included all patients with diagnoses of IBS, FD, or constipation recorded plus a large sample of healthy controls without any diagnosis of a DGBI. ED diagnoses included were anorexia nervosa, bulimia nervosa, and binge ED. Any ED was defined as anorexia nervosa and/or bulimia nervosa and/or binge ED.

### Statistical analysis

After exclusion of any patient in whom an organic GI disorder was identified, a sample of n = 1,256,331 was available (Table 1). The relationship between DGBI and ED prevalence is explored in multiple steps. First the association between specific and then any DGBI is explored by unconditional logistic regression. Odds ratios, with 95% confidence intervals (CIs), representing the association are reported unadjusted and also after controlling for a number of extraneous factors that are known to be related to either or both conditions, anxiety, depression, prolonged proton pump inhibitor (PPI) use (>30 days), and antidepressant use (Table 2). In the second step, patients in whom diagnoses of both DGBI and ED were identified were classified as either having DGBI precede ED or ED precede DGBI. In this step, patients who had a DGBI diagnosed within 30 days of an ED were excluded, yielding a sample of n = 1,018 patients. Moderation of the sequence of presentation of ED and DGBI was assessed by unconditional logistic regression in which the odds of a DGBI diagnosis preceding an ED diagnosis was the outcome (Table 3). Multiple logistic regression was performed to compare a diagnosis of a DGBI first vs ED first on a range of sociodemographic factors (age, sex), medical conditions (anxiety and depression) both any time and antecedent to either a DGBI or ED, and medication use (PPI for >30 days and antidepressants) both at any time and antecedent to either a DGBI or ED. Only individual moderators that reached the criterion of *P* < 0.01 were included in the multivariate model. Finally, in a third step, n = 777 patients in whom diagnosis of all 3 of ED, DGBI, and psychological disorder were identified were used to evaluate characteristics of individuals in whom a given condition was the first identified (Table 4).

**Table 1.** Demographic and psychological characteristics of the study population

	Study group			
	Neither	ED only	DGBI only	Both
N	846,457 (67.4%)	961 (0.1%)	407,874 (32.5%)	1,039 (0.1%)
Age at 1st contact	39.358 (21.802)	22.713 (15.220)	37.847 (18.467)	23.288 (13.113)
Sex				
Female	473,414 (55.9%)	906 (94.3%)	237,446 (58.2%)	975 (93.8%)
Male	373,043 (44.1%)	55 (5.7%)	170,428 (41.8%)	64 (6.2%)
Anxiety				
No	782,513 (92.4%)	697 (72.5%)	331,854 (81.4%)	605 (58.2%)
Yes	63,944 (7.6%)	264 (27.5%)	76,020 (18.6%)	434 (41.8%)
Depression				
No	710,548 (83.9%)	435 (45.3%)	272,067 (66.7%)	312 (30.0%)
Yes	135,909 (16.1%)	526 (54.7%)	135,807 (33.3%)	727 (70.0%)

DGBI, disorders of gut-brain interaction; ED, eating disorder.

**Table 2.** Association of specific DGBI and eating disorders by logistic regression analysis

Eating disorders	IBS (n = 168,678)	FD (n = 260,537)	Chronic constipation (n = 15,230)	Overlap IBS and/or FD^a^
Anorexia nervosa				
Unadjusted OR (95% CI), *P*	1.57 (1.48, 1.67)*	1.92 (1.83, 2.02)**	2.26 (1.96, 2.61)***	1.52 (1.16, 2.10)*
Adjusted OR (95% CI), *P*	1.15 (1.08, 1.22)*	1.64 (1.56, 1.72)*	2.12 (1.84, 2.45)***	1.17 (0.89, 1.53)
Bulimia nervosa				
Unadjusted OR (95% CI), *P*	3.36 (2.89, 3.91)***	1.36 (1.16, 1.60)*	2.12 (1.34, 3.34)***	1.67 (1.25, 2.24)*
Adjusted OR (95% CI), *P*	1.72 (1.47, 2.01)*	1.04 (0.89, 1.23)	1.80 (1.14, 2.83)*	1.21 (0.90, 1.62)
Binge eating				
Unadjusted OR (95% CI), *P*	3.17 (2.48, 4.07)***	1.29 (0.98, 1.68)	1.17 (0.44, 3.15)	1.70 (1.05, 2.75)#
Adjusted OR (95% CI), *P*	1.81 (1.40, 2.33)*	1.04 (0.79, 1.36)	1.03 (0.39, 2.77)	1.27 (0.78, 2.06)
Any eating disorder^b^				
Unadjusted OR (95% CI), *P*	1.73 (1.64, 1.83)*	1.84 (1.76, 1.93)**	2.21 (1.93, 2.53)***	1.66 (1.38, 1.99)***
Adjusted OR (95% CI), *P*	1.53 (1.16, 1.29)*	1.56 (1.48, 1.63)*	2.06 (1.79, 2.36)***	1.24 (1.03, 1.49)#

FD, functional dyspepsia (vs non-FD); IBS- irritable bowel syndrome (vs non-IBS); Constipation (vs no constipation).

CI, confidence interval; DGBI, disorders of gut-brain interaction; OR, odds ratio. Adjusted for age, sex, and psychological disorders.

a Comparison group is having IBS or FD but not both.

b One or more of anorexia nervosa, bulimia nervosa, or binge eating.

#*P* < 0.05, *P < 0.01, **P < 0.001, ***P < 0.0001, and OR>=2.0: a priori defined as clinically meaningful.

**Table 3.** Associations between potential risk factors of an ED diagnosis occurring after diagnosis of a DGBI

Potential moderators of pattern of presentation	DGBI n (%)	ED n (%)	OR (95% CI)	*P* value
Age at first DGBI (mean, SD, yr)	29.1 (13.5)	31.6 (10.9)	0.98 (0.97, 0.99)	<0.001
Sex (female)	382 (92.7%)	572 (94.4%)	1.32 (0.79, 2.19)	0.281
Anxiety at any time	181 (43.9%)	247 (40.8%)	1.14 (0.88, 1.47)	0.314
Depression at any time	291 (70.6%)	425 (70.1%)	1.02 (0.78, 1.35)	0.864
PPI use at any time	238 (57.9%)	334 (55.3%)	1.11 (0.86, 1.43)	0.411
Antidepressant use at any time	363 (88.3%)	503 (83.3%)	1.52 (1.05, 2.19)	0.026
Antecedent anxiety	59 (14.3%)	66 (10.9%)	1.37 (0.94, 1.99)	0.102
Antecedent depression	136 (33.0%)	151 (24.9%)	1.48 (1.13, 1.96)	0.005
Antecedent PPI use	81 (19.7%)	46 (7.6%)	2.98 (2.02, 4.38)	<0.001
Antecedent antidepressant use	163 (39.6%)	175 (28.9%)	1.61 (1.24, 2.10)	<0.001

CI, confidence interval; DGBI, disorders of gut-brain interaction; ED, eating disorders; OR, odds ratio; PPI, proton pump inhibitors.

**Table 4.** The prevalence of anxiety and depression in patients with coexisting eating disorders and DGBI based on the direction of presentation

Variables	Psych 1st	DGBI 1st	ED 1st	*P* values
N	333 (42.9%)	167 (21.5%)	277 (35.6%)	
Anxiety				
No	141 (42.3%)	74 (44.3%)	128 (46.2%)	0.631
Yes	192 (57.7%)	93 (55.7%)	149 (53.8%)	
Depression				
No	15 (4.5%)	20 (12.0%)	15 (5.4%)	0.004
Yes	318 (95.5%)	147 (88.0%)	262 (94.6%)	

DGBI, disorders of gut-brain interaction; ED, eating disorder; Psych, psychological disorders.

Owing to the large sample size and high statistical power for even weak associations, a DGBI was considered to be clinically meaningfully associated with an ED if *P* < 0.01 and OR ≥ 2.0. This criterion was used to avoid erroneously concluding that an association exists based solely on the large sample size available and consequent high statistical power for even trivial effect sizes.

## RESULTS

### Characteristics of the study population

The mean ± SD age of onset of DGBI (37.85 ± 18.47 years) was higher than ED (22.71 ± 15.22 years). In the ED cohort, female sex (94.3%) was substantially higher than male (5.7%), while among the population diagnosed of DGBI, 58.2% were women whereas 41.8% were men (Table 1).

### Prevalence of DGBI, ED, and anxiety and depression

The prevalence of DGBI in the sample (n = 1,256,331) was 13.4% (n = 168,678) for IBS, 20.7% (n = 260,537) for FD, and 1.2% (n = 15,230) for chronic constipation. The prevalence of ED in the studied sample (n = 1,256,331) was 0.6% (n = 7,422) for anorexia nervosa, 0.1% (n = 751) for bulimia nervosa, 0.02% (n = 282) for binge ED, and 0.7% (n = 8,334) for any ED. In the total sample, the prevalence of anxiety and depression was 11% and 22%, respectively, whereas the prevalence of anxiety and depression in the DGBI/ED combined cohort was substantially higher at 42% and 70%, respectively (Table 1).

### Association of ED and DGBI in the study population

IBS was significantly associated with bulimia nervosa and binge ED, whereas FD was significantly associated with anorexia nervosa and any ED in an unadjusted model, but these associations were not also clinically significant (as the OR was < 2.0) after controlling for sex, anxiety, and depression. Chronic constipation was significantly associated with anorexia nervosa, and was also clinically significant for anorexia nervosa, OR = 2.12 (95% CI 1.84, 2.45) and any ED, OR = 2.06 (95% CI 1.79, 2.36) after adjusting for confounders as summarized in Table 2.

### Association of ED with FD and IBS overlap compared with either IBS or FD

The risk of developing anorexia nervosa (OR 1.52, 95% CI 1.16, 2.0 *P* = 0.002), binge ED (1.70 95% CI 1.05, 2.75 *P* = 0.03), bulimia nervosa (OR 1.6, 95% CI 1.25, 2.24, *P* = 0.001), or any ED (OR 1.66, 95% CI 1.38, 1.99 *P*=<0.001) was significantly higher in FD and IBS overlap compared with either FD or IBS, but these associations did not remain clinically significant after controlling for sex, anxiety, and depression (ORs < 2.0, Table 2).

### Order of ED and DGBI diagnoses

We hypothesized a DGBI may predispose to an ED. There were significantly more patients in primary care diagnosed with an ED (59.5%, n = 606) preceding the onset of a DGBI rather than vice versa (40.5%, n = 412, *P* < 0.001). The mean (SD) duration between having an ED precede a DGBI diagnosis was 6.5 (5.3) years, which was longer than for a DGBI diagnosis preceding an ED (5.2 (4.7) years, *P* < 0.001). Equivalent intervals for psychological disorders (anxiety and depression) were 4.9 years (SD 4.2) when ED was diagnosed first, and 5.7 years (SD 4.9) when psychological disorders were diagnosed first (*P* < 0.001).

We found in univariate analyses that younger age of first DGBI (OR = 0.98, 95% CI 0.97, 0.99, *P* = 0.001), greater use of antidepressants at any time (OR = 1.52, 95% CI 1.05, 2.20, *P* = 0.03), antecedent use of antidepressants (OR = 1.61, 95% CI 1.24, 2.10, *P* < 0.001), antecedent depression (OR = 1.49, 95% CI 1.13, 1.96, *P* = 0.005), and antecedent use of PPIs (OR = 2.98, 95% CI 2.02, 4.39, *P* < 0.001) were significantly associated with an increased odds of reporting a DGBI before an ED (Table 3). In a multivariate model, we found younger age of first DGBI (OR = 0.97, 95% CI 0.96, 0.98, *P* < 0.001), antecedent depression (OR = 1.57, 95% CI 1.17, 2.10, *P* = 0.003), and antecedent PPI use (OR = 3.36, 95% CI 2.24, 5.01, *P* < 0.001) to be independently associated with a greater likelihood of a DGBI preceding an ED. We examined whether, among antidepressant medications, selective serotonin reuptake inhibitors, tricyclic antidepressants, or others were differentiated in how they moderated the order of incidence. Although there was some variation, with tricyclic antidepressants being the most strongly associated with DGBI preceding ED, all categories of antidepressant were positively associated with DGBI being diagnosed before ED.

### Order of ED, DGBI, and psychological disorder presentation

**Figure 1.**
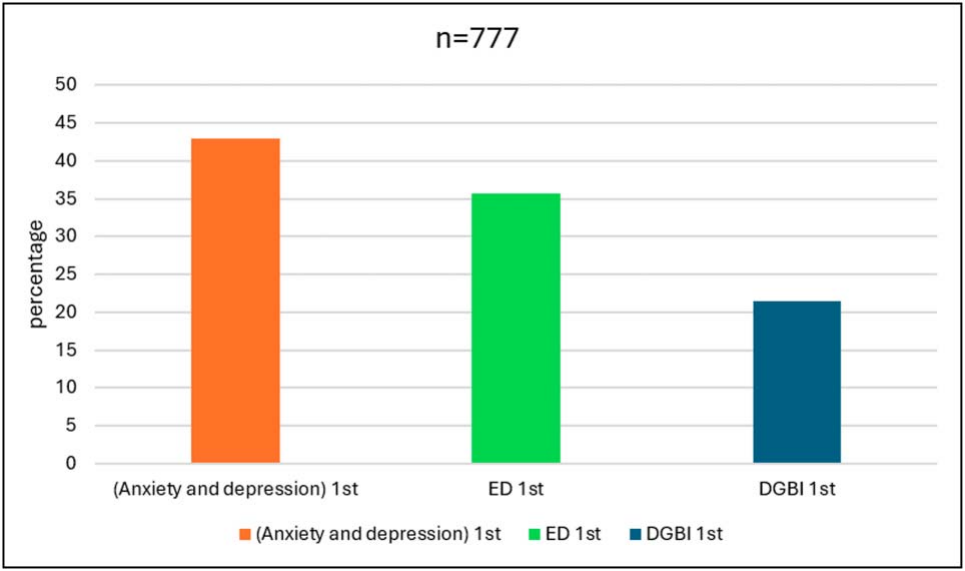
Order of onset of ED, DGBI, and psychological comorbidity. DGBI, disorders of gut-brain interaction; ED, eating disorders.

Of the n = 777 for whom diagnoses of ED, DGBI, and a psychological disorder (anxiety or depression) were identified, we observed 333 (42.9%) reported their psychological disorder first (95.5% had depression whereas 57.7% reported anxiety), 167 (35.6%) were diagnosed of an ED initially, whereas 277 (21.5%) had an initial diagnosis of a DGBI as represented in Figure 1. The prevalence of depression was significantly higher among patients with preexisting ED (94.6%) or DGBI (88.0%) compared with anxiety (53.8% vs 55.7%), respectively (*P* = 0.004, see Table 4).

## DISCUSSION

This study provides new evidence that DGBI can predispose to the development of ED or vice versa, and depression is an independent risk factor of an ED developing in those with a DGBI.

In our study, a diagnosis of chronic constipation was significantly associated with anorexia nervosa and any ED. Anorexia nervosa is an ED characterized by an intense fear of weight gain and a disturbed body image, motivating severe dietary restriction or other weight loss behaviors such as purging or excessive physical activity (17,18). A possible mechanism of constipation after anorexia nervosa is slowing of colonic transit as a sequel to reduced stool bulk formation from reduced intake. This postulation is supported by the normalization of colonic transit time in women with anorexia nervosa after a 4-week refeeding program (19). On the other hand, delayed colonic transit time can induce a reflex slowing of gastric emptying (cologastric brake) potentially further exacerbating symptoms (bloating and fear of weight gain) that could trigger food restriction in anorexia nervosa (20,21). Pelvic floor dysfunction was also reported in 42% of women with anorexia nervosa presenting with chronic constipation (19). Williams et al (22) in a retrospective study of patients with ED found that anorexia nervosa binge-eating/purging subtype reported higher pelvic floor dysfunction than patients with the restricting subtype. They also observed that pelvic floor interventions aimed at improving coordination of the internal pelvic floor muscles improved symptoms of ED (22). However, in a study of patients presenting for anorectal manometry in a tertiary hospital, Murray et al (23) found no differences in anorectal manometry or colonic transit time between patients with anorexia nervosa and control. Future research is therefore required to understand the factors driving the pathogenesis of ED in patients with chronic constipation.

In our study, IBS was associated with bulimia nervosa and binge ED; however, this association did not remain clinically relevant after controlling for confounders (OR <2.0). The use of an OR cutoff of 2.0 in this study to define clinical significance was arbitrary and does not exclude a real albeit modest association between IBS and ED. Similarly, Dejong et al (24) in an outpatient ED clinic found a high prevalence (68.8%) of IBS among patients with bulimia nervosa. Binge eating associated with bulimia nervosa can impair gastric accommodation and further increase intestinal hypersensitivity in IBS (25,26). There is also evidence that binge eating behaviors (changes in dietary patterns, use of laxatives) is associated with alterations in the intestinal microbiome which may in part underly the pathogenesis of IBS (27,28).

FD is a common gastroduodenal DGBI, and the majority have meal induced symptoms of bothersome postprandial fullness or early satiety referred to as postprandial distress syndrome (29). Anorexia nervosa and any ED were associated with FD in the present study, but the OR was below the cutoff point considered clinically meaningful after adjusting for confounders. Meal-related symptoms of FD are common in patients with ED, with most (90%) of the patients diagnosed with anorexia nervosa meeting the postprandial distress syndrome criteria for FD (30). Carpinelli et al (31) in an outpatient ED clinic found 94.7% of an anorexia nervosa cohort met the Rome IV diagnostic criteria for FD of which 89% presented with the postprandial distress syndrome subtype.

Patients with IBS and FD overlap had significantly higher odds of any ED, anorexia nervosa, binge eating and bulimia nervosa compared with either IBS or FD, although this was below the set OR of 2.0. This suggests that the likelihood of disordered eating is increased in DGBI overlap. Patients with overlapping DGBI typically experience more severe GI symptoms and increased psychosocial burden, probably because of shared pathophysiological mechanisms (32,33). The increased symptom burden in multiple DGBI could incite a maladaptive eating behavior that triggers an ED. Wiklund et al (4) in a case-control study found that individuals reporting a high current ED had a higher mean number of DGBI (3.34) compared with those with low current symptoms (1.84). Therefore, clinicians should have a high index of suspicion of ED among patients with multiple overlapping DGBI.

There were significantly more patients in primary care diagnosed with an ED (59.5%) preceding the onset of a DGBI than vice versa (40.5%). We expected ED to result in DGBI symptoms (2). Restrictive eating or starvation can trigger altered GI function and chronic symptoms in many patients. Almeida et al in a retrospective study of patients with a GI encounter in a US hospital observed DGBI were significantly more prevalent in those with preexisting ED (62.5%) compared with those with subsequent ED (42.7%), consistent with our observations (5). In this study, the average duration between an ED preceding a DGBI diagnosis (6.5 years), was significantly longer than a time lag of 5.2 years in cohorts with a DGBI diagnosis predating an ED. Although our findings may not reflect the exact chronology of events because of variations in the onset of symptoms and clinic attendance, it underscores the importance of early screening of DGBI-like symptoms in ED.

On the other hand, we postulated a DGBI may predispose to an ED and this may be driven by comorbid depression or anxiety. The observed association between DGBI and any ED could have been underestimated because ARFID was not coded in the THIN database. Perhaps, if ARFID was included, the reported odds ratio for this association would have approached the preset cutoff value.

The evaluation of the moderators of the pattern of presentation of ED and DGBI showed a younger age of first DGBI, antecedent depression and antecedent PPI use were independently associated with a greater risk of a DGBI preceding an ED diagnosis. Almeida et al (5) among patients with ED attending a GI clinic found younger age at GI consult was associated with detecting a preexisting ED. Because both conditions are more prevalent in the younger population, it is presumed that the age of onset is not affected by the directionality of presentation. Proton pump inhibitors are first-line acid suppressants used to relieve the upper GI symptoms of DGBI, notably FD (34); therefore, it is not surprising that the use of PPI predicted the onset of DGBI before an ED. Furthermore, a recent systematic review reported that prolonged PPI use can be associated with increased risk of small intestinal bacteria overgrowth after gastric acid suppression (35). This alteration in the gut microbiome could possibly lead to the exacerbation of DGBI (36). Although, there is no clear evidence that a PPI use could precipitate an ED, the findings of a subsequent ED in patients on PPI could be linked to changes in eating behaviors because of gut dysbiosis-induced visceral sensitivity and worsening of GI symptoms (36,37).

We have shown affecting on the interplay between ED and DGBI is anxiety and/or depression. The prevalence of anxiety and depression diagnoses in the cohorts with coexisting DGBI and ED in our study were 42% and 70%, respectively. The association between psychological impairment and DGBI/ED coexistence may be explained in part by worsening of the perception of body dysmorphia attributed to the sensation of distension from binge eating (31). The high comorbidity of anxiety and depression in patients with ED or DGBI is detrimental to quality of life and healthcare utilization (13,38). Melchoir et al in a case-control study of 456 patients with IBS in a tertiary care center, using the Hospital Anxiety and Depression scale, found that the prevalences of anxiety (19.0% vs 1.9%) and depression (60.3% vs 19.7%) were significantly higher in IBS patients with ED than in healthy volunteers (39). Other studies showed severe anxiety (77.7%–90%) and depression (25%–95%) were common among patients with coexisting ED and DGBI (13,31). The variations in the prevalence of anxiety and depression could be related to the heterogeneity of DGBI and the differences in the tools used for the assessment of psychological wellbeing.

The onset of anxiety and/or depression, predominantly depression, before ED and DGBI suggests that psychological impairment is a possible driver of both ED and DGBI. The direction of presentation or coexistence of disorders can obscure the distinct clinical manifestations of individual conditions, influencing diagnosis, treatment strategies, and outcomes. This highlights the need for early screening and detection of direction and coexistence of ED or anxiety and depression with DGBI to enhance a multidisciplinary approach to patients' management and improved clinical outcomes (40).

### Strengths of the study

The study was conducted in a large data set representative of the UK general practice with availability of follow-up data to determine the sequence of presentation of the disorders in the population studied. The application of read diagnostic codes provides reassurance the clinical diagnoses are likely to be accurate and consistent (41) and likely minimizes diagnostic bias inherent in using symptom data as a proxy for diagnosis. The use of real-world data provides clinically relevant information.

### Limitations

There is a possibility of a lag between symptom onset and actual diagnosis of DGBI, ED and psychological disorders. This study was unable to assess all ED encountered in clinical practice, particularly avoidant/restrictive food intake disorder. The coding on THIN was recorded using the clinician's diagnosis, which is not explicitly based on Rome criteria or *DSM-V*. The lack of a standardized criteria or precise definition of DGBI or ED could lead to a diagnostic bias, and inaccurate interpretation of findings. For instance, the prevalence of constipation was lower than expected and may have been under-coded or folded into the IBS coding. In addition, there were a limited number of potential moderators of order of onset of ED and DGBI included in our study, and we cannot exclude other factors including dietary restrictions, use of laxatives, severity of GI conditions, or delayed colonic transit time may have influenced the pattern of presentation.

### Future directions

Future research is necessary to understand the mechanisms underlying the complex interplay between ED and DGBI and to ascertain the impact of anxiety and depression on these mechanisms. Researchers need to further explore factors that may influence the bidirectional presentation of ED and DGBI, as the identification and modification of these factors could improve outcomes.

DGBI are associated with ED in primary care patients. An ED more frequently preceded the onset of a DGBI, but younger age of first DGBI, antecedent depression, and antecedent PPI use were independently associated with DGBI presenting before an ED.

## CONFLICTS OF INTEREST

**Guarantor of the article:** Nicholas J. Talley, MD, PhD, MACG.

**Specific author contributions:** N.K.: involved in study conception and design, data analysis and interpretation of results, and manuscript drafting and preparation. S.M.E.: involved in study conception and design, interpretation of results, and manuscript drafting and preparation. M.P.J.: involved in study conception and design, acquisition of data, data analysis, and manuscript drafting. S.A.: involved in interpretation of results and manuscript drafting. H.G.: involved in interpretation of results and manuscript drafting. N.J.T.: involved in study conception and design, acquisition of data, acquiring funding, interpretation of results, and manuscript drafting.

**Financial support:** Funding to obtain access to the data was provided by a grant AusEE Inc (Australia).

**Potential competing interests:** N. Koloski, S.M. Egboh, M.P. Jones, A Shah disclose no conflicts. Prof. N.J. Talley: Brown University, Agency for Health Care Research and Quality (fiber and laxation) (2024), Rome Foundation (member gastroduodenal committee), Biocodex (FD diagnostic tool), Microba (microbiome) Comvita Manuka Honey (FD trial) (2025), BluMaiden (microbiome) outside the submitted work. In addition, Dr. Talley has licensed with MAPI Nepean Dyspepsia Index (NDI) 1998, Licensing Questionnaires Talley Bowel Disease Questionnaire licensed to Mayo/Talley, “Diagnostic marker for functional gastrointestinal disorders” Australian Provisional Patent Application 2021901692, “Methods and compositions for treating age-related neurodegenerative disease associated with dysbiosis” US Patent Application No. 63/537,725. Prof. Gerald Holtmann received unrestricted educational support from the Falk Foundation. Research support was provided by the Princess Alexandra Hospital, Brisbane by GI Therapies Pty Ltd., Takeda Development Center Asia, Pty Ltd., Eli Lilly Australia Pty Ltd., F. Hoffmann-La Roche Ltd., MedImmune Ltd., Celgene Pty Ltd., Celgene International II Sarl, Gilead Sciences Pty Ltd., Quintiles Pty Ltd., Vital Food Processors Ltd., Datapharm Australia Pty Ltd. Commonwealth Laboratories, Pty Ltd., Prometheus Laboratories, FALK GmbH & Co KG, Nestle Pty Ltd., Mylan, and Allergan (prior to acquisition by AbbVie Inc). Dr. Holtmann is also a patent holder for a biopsy device to take aseptic biopsies (US 20150320407 A1).

**Study Highlights**

**WHAT IS KNOWN**

✓ There is an association between disorders of gut-brain interaction (DGBI) and eating disorders. However, there is a gap in ascertaining which of these disorders predates the other and the role of anxiety and depression in the evolution of both conditions.

**WHAT IS NEW HERE**

✓ We found that eating disorders more frequently preceded the onset of a DGBI, and that anxiety and depression are possible drivers of both DGBI and eating disorders, with depression being predominantly reported, irrespective of the order of presentation.
✓ Therefore, this study provides novel information that gut dysfunction is likely a driver of eating disorder in a subset of patients, suggesting early modulation of gut dysfunction in eating disorders could improve management outcomes.

## ABBREVIATIONS:

ARFID: avoidant restrictive food intake disorder
CI: confidence interval
DGBI: disorders of gut brain interactions
ED: eating disorders
FD: functional dyspepsia
HPA: hypothalamus-pituitary-adrenal axis
IBS: irritable bowel syndrome
OR: odds ratio
THIN: The Health Improvement Network
UK: United Kingdom
US: United States
